# Phosphoglycerate Dehydrogenase: Potential Therapeutic Target and Putative Metabolic Oncogene

**DOI:** 10.1155/2014/524101

**Published:** 2014-12-09

**Authors:** Cheryl K. Zogg

**Affiliations:** ^1^Center for Surgery and Public Health, Harvard Medical School and Harvard School of Public Health, Department of Surgery, Brigham and Women's Hospital, 1620 Tremont Street, Boston, MA 02120, USA; ^2^Department of Biochemistry and Molecular Biology, The Johns Hopkins Bloomberg School of Public Health, 615 North Wolfe Street, Baltimore, MD 21205, USA

## Abstract

Exemplified by cancer cells' preference for glycolysis, for example, the Warburg effect, altered metabolism in tumorigenesis has emerged as an important aspect of cancer in the past 10–20 years. Whether due to changes in regulatory tumor suppressors/oncogenes or by acting as metabolic oncogenes themselves, enzymes involved in the complex network of metabolic pathways are being studied to understand their role and assess their utility as therapeutic targets. Conversion of glycolytic intermediate 3-phosphoglycerate into phosphohydroxypyruvate by the enzyme phosphoglycerate dehydrogenase (PHGDH)—a rate-limiting step in the conversion of 3-phosphoglycerate to serine—represents one such mechanism. Forgotten since classic animal studies in the 1980s, the role of PHGDH as a potential therapeutic target and putative metabolic oncogene has recently reemerged following publication of two prominent papers near-simultaneously in 2011. Since that time, numerous studies and a host of metabolic explanations have been put forward in an attempt to understand the results observed. In this paper, I review the historic progression of our understanding of the role of PHGDH in cancer from the early work by Snell through its reemergence and rise to prominence, culminating in an assessment of subsequent work and what it means for the future of PHGDH.

## 1. Introduction

As a genetic disease, cancer is primarily caused by mutations in oncogenes and tumor suppressors, which serve to control tissue homeostasis [1]. Altered function, in turn, leads to deregulated mitogenic survival and growth of tumors that frequently exhibit oncogene-activating genomic alterations such as gene amplification or gain-of-function point mutations. Tumors may also exhibit tumor-suppressor inactivating mutations, including gene deletions, loss-of-function point mutations, or epigenetic silencing [1]. Taken together, these changes enable cells to acquire “stereotypical capabilities” termed by some scholars “hallmarks of malignancy” [1, 2]. Over the past 10–20 years, increasing evidence has shown that the majority of oncogenes and tumor suppressors also play a role in the regulation of metabolism. Mutations serve to “orchestrate nutrient utilization in a manner that facilitates cell survival and growth” [1]—a phenomenon exemplified in the work of Warburg et al. [3, 4].

**Figure 1:**
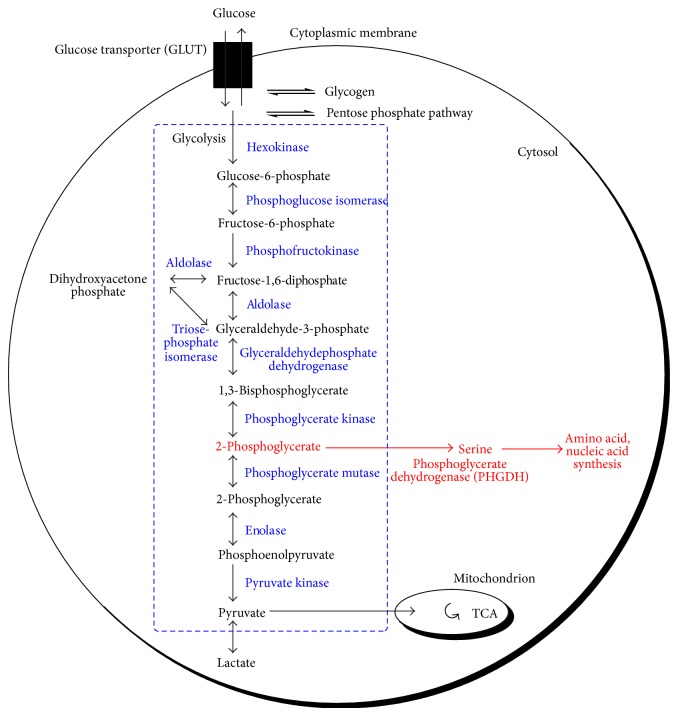
Schematic of glycolysis in a mammalian cell showing a branch point wherein glycolytic intermediate 3-phosphoglycerate can be channeled into a serine biosynthetic pathway via the activity of putative metabolic oncogene phosphoglycerate dehydrogenase (red), adapted from Hamanaka and Chandel [15].

The “Warburg effect” named in his honor describes a process wherein cancer cells preferentially use fermentative glycolysis-based glucose metabolism instead of entering the tricarboxylic acid cycle (TCA) and subsequent electron transport chain, even under aerobic conditions [3–6]. The causes and benefits of the effect have been the focus of study for many years [5, 7], yet despite advances in biochemical understanding, the physiological “reason” remains unclear [6]. One hypothesis suggests that cancer cell proliferation is not limited by ATP production but rather by the ability of cells to synthesize lipids, nucleic acids, and proteins needed to bolster an expanding biomass [6]. Preferential aerobic glycolysis would allow cancer cells to adapt metabolism to satisfy a resultantly increased biosynthetic need—an idea supported by evidence which suggests that the enzyme pyruvate kinase, catalyzing the final step in glycolysis as shown in Figure 1, is inhibited in tumorigenic cells [8]. The observed selection toward reduced pyruvate kinase activity may further enable upstream glycolytic intermediates (e.g., 3-phosphoglycerate) to be diverted into other metabolic pathways used to produce the additional lipids, nucleic acids, and proteins that Mullarky et al. [6] describe [8]. Blockage of the constituent metabolic activities in experimental systems has been found to suppress tumor cell growth* in vitro* and* in vivo* [1].

Metabolic reprograming in tumorigenic cells is not limited to deregulation by oncogenes and tumor suppressors but can also result from genomic modifications to the metabolic enzymes themselves, independently contributing to biomass accumulation and proliferative growth [9–14]. This later class of facilitative oncogenes has started to generate an increasing amount of interest in recent years as researchers return to classic studies to uncover the potential utility of seemingly overlooked historic results and apply them to human tumor growth. It is hoped that, by “shedding light on the biological basis of malignancy,” new applications of old discoveries may lead to the development of novel cancer therapeutics [1]. To be an attractive candidate for cancer therapy, there must be a significant difference between the requirements for a particular enzyme's activity in cancer and normally proliferating cells [15]. It is here that putative metabolic oncogenes become “both interesting and important” given their propensity to display metabolic activities that “differ significantly [and], in some cases profoundly,” from those of enzymes in normal cells [1].

**Figure 2:**
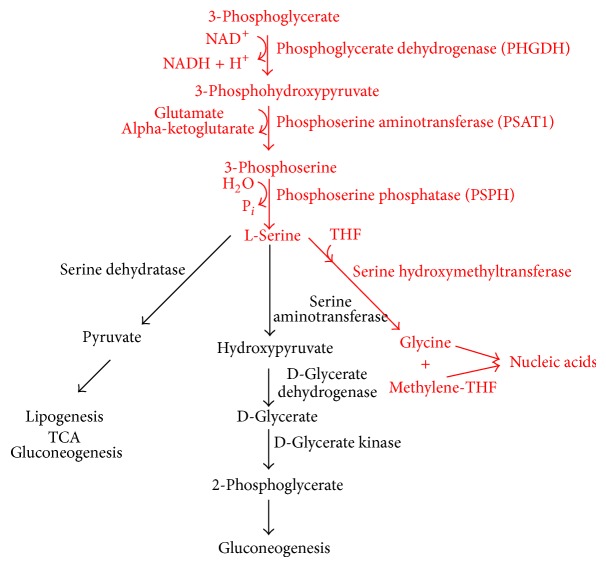
Schematic of the major pathways of serine metabolism in a mammalian liver. Shown in red, phosphoglycerate dehydrogenase, the first enzyme branching from glycolysis in a three-step serine biosynthetic pathway uses NAD^+^ as a cofactor to oxidize 3-phosphoglycerate into phosphohydroxypyruvate. The product is then subsequently converted into phosphoserine via transamination by phosphoserine aminotransferase and, ultimately, to serine via phosphate ester hydrolysis and the enzyme phosphoserine phosphatase. Classic work by Snell [16] suggests preferential upregulation of the serine hydroxymethyltransferase branch, leading to nucleic acid synthesis coupled with downregulation of the serine dehydratase and serine aminotransferase branches in some subsets of cancer cells, adapted from Snell [16].

One example is the conversion of glycolytic intermediate 3-phosphoglycerate into phosphohydroxypyruvate by the enzyme phosphoglycerate dehydrogenase (PHGDH)—a rate-limiting step in the conversion of 3-phosphoglycerate to serine (Figures 1 and 2) [15]. In the paper that follows, I review the historic progression of our understanding of the role of PHGDH in cancer from the classic work of Snell et al. [16–20] in the 1980s through its reemergence and rise to prominence in 2011 with near simultaneous publications by Possemato et al. [21] and Locasale et al. [8], culminating in an assessment of subsequent work and what it means for the future of PHGDH as a potential therapeutic target and putative metabolic oncogene.

## 2. Classic Studies: Animal Models and Early Work by Snell

In a review of serine metabolism published in 1984, Snell [16] provides what is widely regarded as one of the first in-depth discussions of the role of PHGDH in tumorigenic cells. Citing a number of prior laboratory results [27], he notes that the activities of enzymes involved in serine biosynthesis had been previously assessed in rat neoplastic tissue [16]. Specifically, enzymatic activity of PHGDH assayed in four transplantable hepatomas (Morris series: 7794B, 7793, 9121, and 5123TC) was elevated 1.7–10.6 times relative to control livers in rats of the same strain [16, 27]. Reductions in dietary protein induced adaptive increases in PHGDH activity in control animals and healthy portions of the tumor-bearing host liver, presumably due to homeostatic efforts to support diminished nucleic and amino acid supplies, but failed to have any effect on the enzyme in hepatoma cells [27]. Snell [16] hypothesized that the observation may suggest a loss of regulatory function or cancerous enzymatic adaptation. Seemingly hesitant to make such a strong claim, he further noted that the observation may also reflect physiological independence between intramuscularly implanted hepatomas and the innate portal circulation of otherwise healthy rats [16]. In the numerous assays performed by Davis et al. [27] in the Morris Lab, only two involving hepatomas 7793 and 5123TC were the subject of multiple analyses, both of which consistently found elevations of PHGDH relative to healthy liver tissue. In 5123TC it was further shown that the overall rate of serine biosynthesis from carbon-labeled 3-phosphoglycerate closely correlated with the activity of PHGDH, indicating that enhanced activity of PHGDH in the four hepatomas was responsible for increased serine biosynthetic capacity—a conclusion, which Snell [16] suggests, “needs to be treated with caution in view of the above comment on protein content and, in any case, only relates to a very limited number and range of tumors.” In the years that followed, Snell's tone began to change as his own research at the Laboratory for Experimental Oncology at Indiana University School of Medicine led to the first real inclination of just how “limited” PHGDH activity in cancer cells might be.

Two years later, Snell and Weber [18] published a paper entitled “Enzymatic Imbalance in Serine Metabolism in Rat Hepatomas” which showed that the activity of PHGDH was indeed increased in tissues with high cell-renewal capacity in addition to elevations in neonatal and regenerating liver cells. Elevations in hepatomas were markedly higher still (8.8–11.9-fold in slow-growing hepatomas and up to 50.8–75.5-fold in fast-growing hepatomas), suggesting an apparent correlation of PHGDH activity with the tumorigenic rate of growth and an important association with both neoplastic transformation (slow-growth tumor) and progression (fast-growth tumor) [18]. Relative to increased activity in neonatal (10-fold when adjusted for differences in cellularity between adult and neonatal livers) and regenerating liver (2.6-fold) cells, the enhanced activity observed, particularly in fast-growing tumor cells, was thought to point to a specificity in the changes of PHGDH activity in cancer cells. Examination of downstream enzyme activity revealed parallel increases in the activity of serine hydroxymethyltransferase and an absence of serine dehydratase and serine aminotransferase contributions (Figure 2), resulting in preferential shunting of glucose via the serine biosynthetic pathway toward the formation of nucleic acid bases [18]. Such coordinated alterations coupled with the previous work of Davis et al. [27] and Snell [16] would seem to suggest that cancer cells (at least in hepatomas) have induced an enzymatic imbalance to meet tumorigenic needs that places PHGDH in a regulatory position. Capable of diverting glucose-derived carbon toward serine biosynthesis and, by way of hydroxymethyltransferase, toward nucleotide formation, such a change confers a selective growth advantage to neoplastic cells relative to their somatic counterparts [18]. Repeated transplantations of the hepatoma lines* in vivo *did not alter the effect [18], leading to what Snell et al. [19] would later describe as robust evidence for “reprogramming of gene expression” in rat hepatoma cells.

Some 19 months later in July 1987, Snell et al. [19] published again this time looking at the activities of PHGDH and hydroxymethyltransferase during the transition of hepatoma cells from a resting, nonproliferative state to induced proliferative growth. During the different phases of fast-growing hepatoma cells in culture, enzyme activities increased rapidly to reach peaks at 24 h during the early exponential or state-transitional phase and then declined as cells reached higher confluency and entered the plateau phase of growth [19]. The burgeoning hypothesis of coordinated regulation was again confirmed and, in fact, further supported by a series of subsequent experiments in which matched changes in carbon-labeled serine incorporation into nucleic acids were observed [19].

Finally in January 1988, Snell et al. [20] published their fourth and final paper on the role of PHGDH in cancer cells, firmly calling into question Snell [16]'s 1984 supposition on the “very limited number and range of tumors” to which the observed reprogramming of gene expression applied [16, 19, 20]. Their work showed that the patterns of serine metabolism in tumors established in the group's previous studies [16–18]—enhanced PHGDH and serine hydroxymethyltransferase, absent serine dehydratase and serine aminotransferase—were consistent in both a transplantable rat sarcoma model and in human colon carcinoma [20]. In tissues lacking serine dehydratase and serine aminotransferase, such as skeletal muscle where the rat sarcoma model was assayed, preferential reorientation for nucleotide formation cannot be achieved by deletion of nonexistent competing enzymes and must instead rely only on substantially increased activity of serine hydroxymethyltransferase in tumors [17, 20]. This final piece of work becomes significant moving forward, for as Snell et al. [20] importantly note, all of their previous studies relied on transplantable rat tumors in animal model systems. However, “if the patterns are to have significance […], particularly in relation to the development of strategies for enzyme-targeted anticancer drug therapy, then they must also be demonstrated in human cancers” [20]. Much like the results from the rat sarcoma, the activity of PHGDH was found to be elevated 10-fold, and that of serine hydroxymethyltransferase nearly 5-fold, in tumors relative to healthy colon mucosa. Competing enzymes of serine utilization were absent from both [20].

## 3. Expression in Humans

Following Snell et al.'s final publication in 1988 [20], the group's work shifted to consider the therapeutic role of serine hydroxymethyltransferase, leaving the discussion of PHGDH all but abandoned for more than a decade. It is not until the work of Cho et al. [22] published in March 2000 that its potential utility as a metabolic oncogene begins to appreciably reemerge. Building on the need to establish expression in human cancer cells, Cho et al. [22] looked at nucleotide sequence and differential expression of the human* PHGDH* gene. Citing work by Achouri et al. [28] that reported successful cloning of the first mammalian* PHGDH* gene from a rat hepatoma in 1997, Cho et al. [22] used 300 cDNA clones randomly sequenced from a *λ* ZAP human Jurkat T-cell cDNA library to look for similarity to the known rat* PHGDH* gene. One 852 bp clone showed partial similarity to the 3′-region and was subsequently used to rescreen the library as a novel probe [22]. Of five hybridization-positive clones, one contained an insert 2,478 bp long. Comparison to other known* PHGDH* sequences and 3D modeling allowed for the assignment of substrate-binding, nucleotide-binding, and regulatory domains. The overall sequence was found to share 94% homology with rat* PHGDH* [28] and 93% homology with murine* PHGDH* [22].

When the sequence was compared via individual functional domains, the nucleotide-binding domain, containing a consensus Gly-Xaa-Gly-Xaa-Xaa-Gly-Xaa_17_-Asp sequence known to be involved in binding the adenosine portion of NAD^+^, showed the highest degree of homology [22]. The C-terminal regulatory domain was more variable, particularly in length, but did not contain alterations considered important for allosteric control [29, 30].

Tissue distribution of PHGDH-specific mRNA was then analyzed using a ^32^P-labeled version of the 2,478 bp PHGDH cDNA probe on a human multiple tissue northern blot assay [22]. Two transcripts, approximately 2,100 bp and 710 bp in size, were detected. The dominant 2.1 kb mRNA transcript was found at high levels in the prostate, testis, ovary, brain, liver, kidney, and pancreas. Lower levels were found in the mucosal lining of the colon and, weakly, in the thymus, small intestine, and heart [22]. In contrast, Achouri et al. [28] found a single 2.1 kb mRNA derived from the liver of a rat detectable only under conditions of a protein-free, carbohydrate-rich diet. Pointing to this discrepancy, Cho et al. [22] note that dietary regulation akin to that mentioned by Snell in 1984 [16] may not be significant in human tissues given that the 2.1 kb mRNA was found expressed at high levels in the human liver even under normal conditions. The secondary 710 bp mRNA was detected as a minor transcript in most of the tissues where the dominant transcript was observed with notably higher levels relative to the dominant transcript in the heart. It was the only transcript found in human skeletal muscle [22]. Taken together, the normal physiological findings of Cho et al. [22] suggest that expression of human PHGDH is not limited to tissues with a high proliferative capacity but rather expressed in a tissue-specific manner. The minor 710 bp mRNA transcript is thought to potentially reflect differential splicing or use of a secondary internal (2.1 kb intron-contained) transcriptional start [22].

Cho et al. [22] then sought to determine what happens to the differential expression of PHGDH-specific mRNA in malignant human tumor cells and whether the anticipated elevation in PHGDH protein expression is due to upregulation of PHGDH mRNA at the transcriptional level or is instead the result of modifications in enzymatic characteristics such as *K*_*m*_, *V*_max⁡_, and/or stability. Akin to their analysis of normal tissue, Cho et al. [22] used a northern blot assay with PHGDH cDNA to reveal that the dominant 2.1 kb mRNA transcript was detectable in most continuously growing tumor cells. PHGDH transcriptional expression was identified in several human leukemias (Jurkat, MOLT-3, HL-60, U937, and THP-1), T-cell lymphoblastic lymphoma (Sup-T1), colon adenocarcinoma (COLO 320DM), epithelioid carcinoma (HeLa S3), and murine lymphoma (BW5147.G.1.4) [22]. The highest levels of expression were observed in COLO 320DM and BW5147.G.1.4. The physiological role for the minor transcript remains unclear, but, of note, its transcriptional expression in human tumors paralleled that of the dominant transcript [22]. On the whole, the results of Cho et al. [22] establish that upregulation of PHGDH at the transcriptional level may be responsible for the elevated enzymatic activity reported in neoplasia by Snell et al. [18–20] and suggest that their assertion of application to human expression can be generalized to include most leukemias and lymphomas of human and murine origin.

## 4. Reemergence of Phosphoglycerate Dehydrogenase: Breast Cancer

Studies of human PHGDH began to appear in subsequent years, prior to prominently reemerging in 2011 with the work of Possemato et al. [21] and Locasale et al. [8]. However, prior to their respective publications in* Nature* and* Nature Genetics*, the work of Pollari et al. [23] set the stage. Metastatic transcriptional expression revealed by Cho et al. [22] led to the identification of enhanced serine production in bone metastatic breast cancer cells and simulation of osteoclastogenesis [23]. Subsequent (and prior) contributions are summarized in Table 1.

Highlighting the incurable nature and significant morbidity of bone metastatic disease, Pollari et al. [23] used breast cancer cell line MDA-MD-231 (derived from an estrogen receptor-negative strain) to model breast cancer bone metastasis* in vivo* via intracardiac inoculation of immunodeficient mice. Comparison of parental MDA-MD-231 and an enhanced daughter variant with heightened metastatic abilities, MDA-MB-231(SA), revealed genetic aberrations that were highly conserved between the two lines [23]. Genome-wide expression profiles pointed to mere 315 genes (1.7% of genes) that were more than 2.5-fold upregulated and 198 genes (1.1% of genes) that were more than 2.5-fold downregulated in the highly metastatic variant relative to the parental strand. Purported pathway associations for the upregulated genes pointed to changes in organic acid, amino acid, and amine metabolism in addition to alterations to nitrogen compound biosynthetic processes—the most significantly enriched of which included the pathway for glycine, serine, and threonine metabolism. Three genes and their corresponding enzymes are involved in the processes of all three: PHGDH, phosphoserine aminotransferase (PSAT1), and phosphoserine phosphatase (PSPH) (Figure 2). Relative to the parental metastatic cell line, highly metastatic MDA-MB-231(SA) daughter cells exhibited upregulations of 5.1-, 5.8-, and 2.6-fold, respectively. A serine/alanine/cysteine/-threonine transporter known as SLC1A4 was likewise elevated 3.4-fold with overexpression of each confirmed by quantitative RT-PCR (mRNA) and Western blot (protein) assays. Subsequent statistical quantification using repeat probe sets further showed that while PHGDH expression was significantly higher (*P* < 0.001) in strongly versus weakly metastatic cells, PSAT1 expression was not significant and PSPH failed to correlate with metastatic ability at all [23].

Pollari et al. [23] concluded their analysis with an assessment of PHGDH, PSAT1, and PSPH expression relative to time to relapse in 368 human clinical breast cancer sample cells as well as relative to overall survival time in 393 breast cancer sample cells. Their secondary analysis presents the first well-documented assessment of clinical outcomes associated with serine biosynthetic elevation in cancer cells. What they found was a statistically significant association between high PHGDH expression and shorter time to relapse (*P* < 0.001) in addition to shorter overall survival time (*P* = 0.002). Further assessment for “clinically relevant features” in a subset of 251 samples pointed to additional associations between enzymatic PHGDH and PSAT1 expression and several recognized risk features, including estrogen and progesterone receptor negative status, mutated p53, higher tumor grade, heightened expression of the cell proliferation markers PCNA and Ki-67, and higher levels of ERBB2 [23].

In August 2011, Possemato et al. [21] published a letter in* Nature*. With the stated aim of identifying metabolic genes required for tumorigenesis, Possemato et al. [21] cross-referenced known maps of metabolic pathways with the Kyoto Encyclopedia of Genes and Genomic (KEGG) database to compile what they considered a “comprehensive list” of 2,752 genes encoding “all known human metabolic enzymes and transporters.” Identified genes were scored on 3 criteria to identify a “high-priority” set of 133 metabolic enzyme and transporter genes: (1) known to have high expression in tumors versus normal tissue, (2) known to have high expression in aggressive breast cancer, and (3) known to be associated with the stem-cell state. Selected candidates had to fit two of the three categories or be “at the top” of a given category. Among the chosen 133 genes, Possemato et al. [21] constructed lentiviral short hairpin RNA (shRNA) vectors targeting the respective genes with a median of 5 shRNA per gene and used them to generate 2 libraries—1 targeting transporters and control genes (235 distinct shRNA) and 1 targeting metabolic enzymes and control genes (516 distinct shRNA). When the libraries were screened for shRNA that became depleted during breast cancer tumor formation in mice, 16 genes “hit” with at least 75% of the corresponding shRNA targeting the respective genes. Expected targets (e.g., the mitochondrial ATP transporter VDAC1) “hit” as did genes involved in control of oxidative stress, the pentose phosphate pathway, glycolysis, proline biosynthesis, and serine biosynthesis. For 5 of the 16 original hits (including PHGDH), 2 of the “scoring” shRNA were tested for their effect on tumorigenesis. Each suppressed target expression in tumor forming breast cancer cultured MCF10DCIS.com cells and reduced the cell's tumor-forming capacity [21].

Consultation of cancerous somatic genome-wide copy number alterations reported in the work of Beroukhim et al. [31] in an effort to prioritize genes revealed that* PHGDH* exists in a region of chromosome 1p commonly amplified in several types of cancer, including cancers of the breast and skin (melanoma) [21]. None of the other hit genes coincided with genomic regions of focal and recurrent copy number gain. Moreover, three shRNA that scored in the* in vivo* screen also decreased PHGDH protein expression; two of differing knockdown efficacies inhibited tumor growth [15].

The initial results of Possemato et al. [21] corroborate the findings of Pollari et al. [23] who, as previously described, reported elevated PHGDH mRNA levels in estrogen receptor-negative breast cancer cells. Cognizant of these findings, Possemato et al. [21] confirmed Pollari et al.'s [23] results in a distinct gene expression set before going on to find that PHGDH was also elevated in estrogen receptor-negative breast cancer relative to normal breast tissue. In an effort to situate these observations within their larger body of work, Possemato et al. [21] note that, of all the genes identified as hits in their previous screen,* PHGDH* had the most significantly elevated expression in estrogen receptor-negative breast cancer cells. Among 82 human breast tumor samples assessed in an immunohistochemical assay (not discussed in this review) PHGDH protein levels significantly correlated with estrogen receptor-negative status [21]. In what became one of the paper's most heavily cited results, Possemato et al. [21] conclusively state that, relative to estrogen receptor-positive breast tumors, estrogen receptor-negative tumors have approximately 68% and 70% elevations of PHGDH, respectively, at the mRNA and protein levels, accounting for an estimated 20–25% of prevalent breast cancer cases and as much as 50% of breast cancer deaths within 5 years of diagnosis [32].

Turning to yet another line of evidence, the letter by Possemato et al. [21] continues to note that, across a set of 8 breast cancer cell lines, 4 with copy number amplifications of* PHGDH* had 8–12-fold higher PHGDH protein expression relative to nontransformed cell lines (lacking gene-based amplification). Where the subsidiary analysis becomes interesting is in its secondary observation that PHGDH protein levels were elevated in two estrogen receptor-negative cell lines, lacking a* PHGDH* copy number gain [21]—a finding which suggests that additional mechanisms beyond genomic amplification must exist to promote upregulation of PHGDH expression at reported mRNA and protein levels.

To better understand the metabolic consequences associated with such increased PHGDH expression, Possemato et al. [21] used metabolite profiling and an analysis of serine synthesis pathway flux to examine breast cancer cells with and without PHGDH genomic amplification. Cell lines with copy number gains (BT-20, MDA-MB-468, and HCC70) experienced increased flux through the serine biosynthetic pathway (Figure 2) relative to those without MDA-MB-231, MCF7, and MCFC10A. Moreover, cell lines with elevated PHGDH and high pathway flux were capable of enhancing proliferation in medium lacking serine, while lines with lower levels of PHGDH underwent significant blunting or cessation of proliferation. Notably, RNAi-mediated suppression of PHGDH resulted in reduced serine pathway flux in both MDA-MB-468 (no amplification) and BT-20 (amplification) cells. Conversely, in MCF10A human mammary cells engineered to overexpress PHGDH, serine pathway flux increased to levels similar to those seen in constitutively amplified cells. Even in the absence of serine, the additional overexpression of PHGDH in modified MCF10A cells was sufficient to drive glucose-originating flux through the biosynthetic serine pathway [21].

In their penultimate experiment, Possemato et al. [21] sought to determine whether PHGDH is required for cell survival and growth in cells exhibiting increased PHGDH expression. They observed that, in cells with high PHGDH expression (whether or not they had copy number mutations), RNAi-mediated suppression of PHGDH caused an appreciable decrease in cell number and induced cell death in the absence of apoptotic markers. In cells with established tumorigenic growth, doxycycline treatment of inducible shRNA reduced PHGDH protein levels in murine mammary fat pad tumors established with transduced MDA-MD-468 cells at 25 days, indicating that PHGDH suppression can adversely affect growth among established tumor cells [21].

Such seemingly contradictory results with respect to serine flux presented a conundrum for Possemato et al. [21]. On the one hand, serine, as shown in Figure 2, is a central metabolite in biosynthetic reactions for amino and nucleic acid production; heightened expression of PHGDH in cancer cells had been found to significantly enhance biosynthetic serine pathway flux. On the other hand, the work of Possemato et al. [21] thus far had shown that PHGDH suppression also inhibited proliferation in cells growing in media with normal levels of extracellular serine (an amino acid which can also be taken up). Supplementation with additional serine or cell-permeable methyl-serine-ester did not blunt the suppressive effect. Looking first for more obvious solutions, Possemato et al. [21] confirmed that intra- and extracellular serine were in equilibrium and that import of extracellular serine was not defective in the cell lines considered. Then, lacking an alternative explanation, they concluded that, perhaps, serine production is not the only important role for PHGDH in cancer cells [21].

Thus, in their final experiment for the paper, the researchers sought to test the notion that PHGDH, PSAT1, and PSPH reactions may produce metabolites beyond serine critical for cell proliferation [21]. They hypothesized that, in cells with high PHGDH expression, the subsequent PSAT1 reaction might contribute a significant fraction of glutamate to alpha-ketoglutarate (aKG) flux—a notion supported by their finding that the serine pathway produces equimolar amounts of serine and aKG. If true, as will be discussed in a subsequent section of the paper, serine biosynthesis would play an important role in anaplerosis of glutamine-derived carbon to the TCA. Consistent with this possibility, suppression of PHGDH in MDA-MB-468 cells caused a large reduction in levels of aKG. Moreover, of the major metabolites measured, aKG had the most significant and largest change upon PHGDH suppression, while serine levels were not significantly affected. Confirmatory labeling studies using U-^13^C-glutamine further revealed that absolute flux from glutamine to aKG and to other TCA intermediates was significantly reduced in cells with RNAi-mediated suppression of PHGDH [21]. In cells with high PHGDH expression, the serine biosynthetic pathway may be responsible for as much as 50% of the net conversion of glutamate to aKG. Suppression of PHGDH would then result in a significant loss of TCA-intermediate flux. Parallel labeling studies in cell lines with PHGDH amplification relative to those without found that flux through the serine biosynthetic pathway shunts 8-9% of glycolytic flux toward serine production as compared to 1-2% in cell lines with low PHGDH expression [21]. Based on this body of evidence, Possemato et al. [21] believe that increased flux through the serine biosynthetic pathway has a major impact on aKG production but a smaller effect on glycolysis and serine bioavailability to tumorigenic cells (at least in the breast) than previously observed.

## 5. Extension to Other Cancers

In their nearly concurrent publication in* Nature Genetics* addressing PHGDH expression in melanoma cells, Locasale et al. [8] challenge the conclusions of Possemato et al. [21], suggesting that PHGDH directly diverts glycolytic flux, thereby, contributing to oncogenesis in cancer cells. Rather than a genomic database, Locasale et al. [8] began their investigation by monitoring the time course of conversion of U-^13^C-glucose using targeted chromatography and mass spectrometry in HEK293T cells. Labeled glucose was incorporated into 13 metabolites across multiple pathways over a 30 min span [8]. Importantly, flux to phosphoserine reached steady state on a time scale similar to that of phosphoenolpyruvate (Figures 1 and 2), suggesting comparable relative flux. ^13^C-phosphoserine labeling paralleled ^13^C-serine labeling—data further corroborated by NMR experiments which indicated that a “substantial fraction” of glucose is diverted from 3-phosphoglycerate toward the conversion of serine and glycine in these cells. To measure the total amount of glucose-derived serine being made, Locasale et al. [8] cultured cells in uniformly labeled ^13^C-glucose and measured metabolites in cell extracts using targeted chromatography and mass spectrometry. Labeled serine accounted for about one-half with corresponding amounts of glucose incorporation detected in subsequent nucleotide and nucleoside intermediate formation. Expression of PHGDH was verified by Western blot; Locasale et al. [8] reported an approximately 30-fold increase in protein expression relative to nontumorigenic cells (absent in MCF10a cells).

Indication of selective glucose diversion led to the notion that there may be a context in which pressure exists for tumors to increase PHGDH activity [8]; much like Possemato et al. [21], Locasale et al. [8] wondered whether the mechanism might involve genomic amplification (a copy number gain) at the locus containing the* PHGDH* gene. To that end, they identified* PHGDH* in the work of Slamon et al. [33] and noted that* PHGDH *was found in a region of chromosome 1p (1p12) known to exhibit recurring copy number gains in 16% of all cancers [8]. Further inspection of the genomic region encoding* PHGDH* revealed localized amplification within the coding region of the gene. In an effort to verify these findings, the researchers examined focal copy number gain using fluorescence in situ hybridization (FISH) with an esophageal squamous cell carcinoma line (T.T. cells) known to contain amplification of* PHGDH*. Stable PHGDH knockdown using shRNA reduced the proliferation rate. Moving on to test whether the decreased proliferation was due to reduced ability to utilize the serine biosynthetic pathway, Locasale et al. [8] generated cell lines with reduced expression of PSAT1 and PSPH to find that shRNA-mediated knockdown of these enzymes resulted in similarly diminished proliferation rates.

Echoing the need for human data first espoused by Snell [16] and Cho et al. [22], Locasale et al. [8] returned to their* PHGDH* amplification results and noted that since amplification in a single tumor type was most commonly found in melanoma, it may be of use to consider PHGDH expression and copy number gain in human melanoma tissue samples. To that end, they used immunohistochemistry to measure PHGDH expression and found that high expression (defined by an IHC score > 1) was observed in 21% of samples. Corresponding copy number gains were detected with FISH in a subsample of 21 out of 42 tumors assayed. To investigate whether melanoma cell lines containing* PHGDH* amplification would be sensitive to decreased expression of PHGDH, cell lines with and without copy number gains were assessed with a methodology similar to that of the studies conducted by Possemato et al. [21]. Amplified cell lines revealed decreased proliferation in response to reduced PHGDH, while nonamplified lines did not change on PHGDH knockdown. Verification of serine metabolic flux further showed that each amplified line did contain appreciable conversion of labeled glucose to serine. PHGDH knockdown reduced phosphoserine levels in Sk-Me128 cells and globally altered metabolite levels, including those of glycolytic intermediates. More specifically, under PHGDH knockdown conditions, heighted glycolytic metabolite levels were detected near the point of diversion onto the serine biosynthetic pathway—the results which Locasale et al. [8] took to mean that the level of PHGDH expressed in a cell alters glucose metabolism (in Sk-Mel128 cells) by modulating entry of glycolytic intermediates into serine metabolism.

However, while copy number gain offers one mechanism to divert flux into serine biosynthesis, Locasale et al. [8] also note that other mechanisms to elevate PHGDH expression likely exist and may be important in specific cancer contexts. For example, pointing to the work of Possemato et al. [21] which found that high PHGDH mRNA was associated with poor prognosis in breast cancer, Locasale et al. [8] undertook a bioinformatics analysis of multiple tumor microarray datasets in breast cancer and found strong associations (*P* < 0.0001) with several clinical parameters. In an effort to validate and expand these results, Locasale et al. [8] assessed PHGDH protein expression in 106 breast cancer tumor samples using immunohistochemistry correlated with mRNA expression to find that high PHGDH expression associated significantly with triple-negative (*P* = 0.002) and basal subtypes (*P* = 0.004) but did not associate with general parameters such as metastasis (as was previously reported) or with tumor size. Consistent with a reliance of a subset of breast cancers on PHGDH, protein expression was required for growth in a panel of three (BT-20, SK-BR-3, and MCF-7) breast cancer cell lines (including the BT-20 cell line that carries amplification). Reduced PHGDH expression decreased phosphoserine levels in PHGDH amplified BT-20 cells, while nontumorigenic breast cancer epithelial MCF-10a cells did not require PHGDH for growth, exhibit alterations in glycolysis on shRNA knockdown of PHGDH, or show detectable labeling of phosphoserine from glucose [8].

Given the importance granted to these two papers and the dense nature of the results that they report, it seems prudent to take a moment to review. Both studies published in mid-2011 report that the gene encoding PHGDH is amplified in a significant subset of human tumors and underscore that diversion of glycolytic intermediates into the serine biosynthetic pathway may contribute to tumorigenesis in cancer cells [5, 8, 21, 34]. Convergence on PHGDH happened through different means in different subsets of cancer-derived laboratory cells, leading to different understandings of the mechanisms involved. Nevertheless, whether using functional genomics or metabolomics methods via Possemato et al. [21]'s loss-of-function RNA-interference screen or Locasale et al. [8]'s glucose-derived carbon flux, both groups identified purported targets of interest and mined known databases of cancer copy number alterations to determine that* PHGDH* on chromosome 1p12 is amplified in some 6% of breast cancers and 40% of melanomas. Beyond genomic amplification, a larger fraction of tumors were found to have elevated PHGDH protein levels, including 70% of estrogen receptor-negative breast cancers. High PHGDH expression (with or without genomic amplification) was associated with dependence on the enzyme for growth either via serine utilization or a hypothesized benefit of aKG to the TCA—a phenomenon that Deberardinis [5] and Seton-Rogers [34] describe as tumorigenic “addiction” to “flexible flux” [5, 8, 21, 34].

In a series of papers that followed Liu et al. [24], Jing et al. [25] and Noh et al. [26] extend these ideas to consider PHGDH's purported role in astrocytoma, cervical cancer squamous cell carcinoma, and triple negative breast cancer cells. Citing the work of Possemato et al. [21] and Locasale et al. [8], Liu et al. [24] sought to determine whether the effect also existed in glioma cells. Analysis of PHGDH levels in specimens from glioma patients revealed that although PHGDH is not normally expressed in healthy brain tissue, significant elevations were observed in astrocytic tumors in increasing correlation with progressively advanced tumor grade. Mechanistic investigations revealed that inhibition of PHGDH expression in glioma cells impaired proliferation, invasion, and tumorigenicity* in vitro* and* in vivo*. In nude mice injected with stable, PHGDH shRNA-silenced glioma cells, overall survival was prolonged relative to mice injected with wild-type cells. The oncogenic transcription factor FOXM1 was also downregulated in PHGDH shRNA-silenced glioma cells. Using liquid chromatography/liquid chromatography-mass spectrometry, Liu et al. [24] identified PHGDH as a novel binding partner for FOXM1. The interaction stabilized FOXM1 protein levels and sequentially induced the expression of a series of oncogenes, including MMP-2, VEGF, Chk2, and cyclin D1—a combined body of evidence which may suggest additional roles for PHGDH in glioma tumorigenesis beyond serine biosynthetic flux akin to that described for aKG in a subset of breast cancer cells by Possemato et al. [21] and, in this case, potentially beyond metabolic functions themselves [24].

The work of Jing et al. [25] follows a similar trend highlighting the role for PHGDH in cervical cancer. As with tumorigenic breast cancer, melanoma, and astrocytoma cells, PHGDH was more strongly expressed in cervical cancer relative to normal cervical epithelium (72 versus 29%, *P* < 0.05) [25]. Expression levels positively correlated with serum squamous cervical cancer antigen in squamous cell carcinoma, a form of cervical cancer (*P* < 0.05)—both of which also associated with tumor progression, stage, and size (*P* < 0.05) [25]. Finally, the work of Noh et al. [26] published in January 2014 echoes the work of Pollari et al. [23] with estrogen receptor-negative breast cancer cells (later corroborated and expanded in the work of Possemato et al. [21]). Using six different subtypes of triple negative breast cancer (TNBC), Noh et al. [26] assembled microarrays of formalin-fixed and paraffin-embedded tissue from 129 TNBC patients. Immunohistochemistry assays for enzymes of serine (and glycine) metabolism, including PHGDH, PSAT1, PSPH, and serine hydroxymethyltransferase, and surrogate markers for identification of molecular tumor type revealed that, among TNBC tumors, basal marker-positive patients exhibited increased expression of PHGDH relative to basal marker-negative patients (*P* = 0.029). On the whole, protein expression of PHGDH tended to be high in patients classified as mixed or basal-like subtype and low in patients classified as immune-related, molecular apocrine, or null subtype but was not statistically different when considered across all six TNBC types (*P* = 0.070). Among mixed subtype cases, 89.3% showed partial expression of basal markers in their mix [26].

## 6. Physiological Relevance of the Findings

Historic progression aside, the question remains “What do these findings actually mean for the utility of PHGDH in cancer cells?” The answer is not clear, rendering PHGDH a fascinating and, at first glance, unintuitive metabolic target in cancer [5]. Known to catalyze entry into a “metabolic side street, diverting flux away from the “superhighway” of tumor cell glycolysis,” it would appear to reduce energy formation from glucose in rapidly proliferating cells [5], yet as the works of Warburg et al. [3, 4] and others have shown, cancer metabolic pathophysiology is seldom so simple. PHGDH copy number, transcriptional, and protein amplifications have been shown in multiple subsets of cancerous cells, clearly indicating that some selective advantage is being attained. While the precise nature of that advantage remains unclear and, in fact, may differ across types of cancerous cells, several hypotheses have been put forward. Metabolic pathways downstream from serine metabolism contribute to growth, promoting biosynthesis and metabolic signaling involving the folate pool, amino acid/lipid intermediates, and redox regulation [8]. The process of diverting glycolytic flux out from 3-phosphoglycerate serves to further alter cellular redox status via the oxidation of 3-phosphoglycerate and to aid in the generation of aKG from glutamate—all of which are reported to benefit cellular proliferation [8].

Considered in more detail, the first and perhaps most readily apparent benefit involves the products of serine biosynthesis itself—*de novo* production of serine and, by way of serine hydroxymethyltransferase, glycine from which multiple amino and nucleic acids can be made. What is interesting about this aspect of the pathway is that cells grown in standard* in vitro* culture conditions consisting of abundant extracellular serine have been found to be sensitive to* PHGDH* knockdown [6]. Perhaps, as Mullarky et al. [6] suggest, cells do not express the correct amino acid transporters to import enough serine or perhaps homeostatic mechanisms regulating metabolic flux are not coupled to extra- and intracellular serine pools. Whatever the reason,* de novo* synthesis of an otherwise nonessential amino acid preferentially occurs such that in its absence cell proliferation suffers or does not occur. This notion of dependency or “addiction” to a “flexible flux” [5, 34] leads to several of the other hypothesized reasons and, consequentially, purported benefits that heighted expression of PHGDH is likely to confer.

Tying into the conversion of serine to glycine by the enzyme serine hydroxymethyltransferase, the second notion stems from recognition that this conversion provides a major source of methyl groups for the one carbon pools required for biosynthesis and DNA methylation [5, 6]. By contributing one-carbon units to the folate pool, serine biosynthesis regulated by PHGDH effectively provides skeletons for the synthesis of purines and pyrimidines (Figure 2), a consequence that led Mullarky et al. [6] to argue that the numerous resultant contributions of PHGDH to biomass production may help to explain part of its protumorigenic effect. The notion is not entirely new. The work described by Pollari et al. [23] found that, in breast tumors, expression of multiple enzymes along the serine/glycine biosynthetic pathway was associated with metastasis in mice and poor clinical outcomes in humans. Moreover, as noted by DeBerardinis [5] and shown by Nikiforov et al. [35] two isoforms of serine hydroxymethyltransferase act as transcriptional targets for the oncogene* c-Myc* with serine hydroxymethyltransferase overexpression leading to stimulated proliferation in* c-Myc-*deficient cells. At the same time, both serine and glycine are abundant in the plasma, so as noted previously by Mullarky et al. [6], it is not immediately apparent what can be gained by upregulating synthesis at the expense of glycolysis, despite the observation that that is precisely what some cancerous cells seem to do [5].

Perhaps the role of aKG used by Possemato et al. [21] to explain their seemingly counterintuitive observation that silencing PHGDH expression failed to deplete intracellular serine can offer insight to the benefit gained. As mentioned above, aKG is a metabolite produced via the transamination reaction of phosphoserine aminotransferase during the conversion of phosphohydroxypyruvate to phosphoserine (Figure 2). It is also the entry point through which glutamine supplies carbon to the TCA cycle during cell growth, thereby enabling production of many essential biosynthetic precursors [5]. As noted by Possemato et al. [21], as much as half of all glutamine-derived aKG in PHGDH-overexpressing cells was generated via an offshoot of the PHGDH-regulated serine biosynthetic pathway. The compelling implication, supported by Possemato et al. [21], is that PGHDH could act as a metabolic gatekeeper, aiding proliferative control of both macromolecular biosynthesis downstream of glutamine metabolism (cell growth) and serine-dependent DNA synthesis (cell proliferation) [5]. Furthermore, as outlined by Mullarky et al. [6], generation of aKG through the serine biosynthetic pathway may help to alleviate ammonia toxicity stemming from amino acid catabolism. aKG synthesis via the serine biosynthetic pathway bypasses a mechanism wherein glutamate derived from glutamine is catabolized by glutamate dehydrogenase to eventually derive aKG, producing a unit of ammonia in the process [6]. Ammonia levels regulate autophagy, and, as Mullarky et al. [6] attest, it would be interesting to see whether cancer cells requiring PHGDH to function have defects in autophagy after PHGDH knockdown.

The suggestion that aKG synthesized from serine is critical for tumor growth is not easy to understand from a metabolic point of view given the numerous potential physiological sources of the compound [36]. It is generated from transamination of a variety of amino acids and is synthesized directly in the TCA cycle. Mullen and DeBerardinis [1] seemingly agree, stating that cancer cells (at least in culture) express a number of other highly active transaminases in addition to PSAT1 which often account for the majority of glutamine-derived aKG. It will be interesting to see as future studies unfold why a subset of cancer cells appear to preferentially use PSAT1 as a source for aKG. As for anaplerosis propagating the TCA cycle, it is a role typically filled by conversion of pyruvate to oxaloacetate (major reaction involving formation of HCO_3_^−^) and the reversible transamination of aspartate to form oxaloacetate (including generation of glutamate and depletion of aKG) coupled with the oxidation of glutamate back to aKG (glutamate + NAD^+^ + H_2_O → NH_4_^+^ + aKG + NADH + H^+^) or the conversion of propionyl-CoA to succinyl-CoA (propionyl-CoA + ATP + HCO_3_^−^  → succinyl-CoA + ADP + P_*i*_) in the *β*-oxidation of fatty acids [36]. Nevertheless, in spite of the questions that remain and the need for detailed pathways to be satisfactorily drawn out, even Kalhan and Hanson [36] acknowledge that “it is clear serine is a major amino acid in the overall metabolism of a number of tumor-derived cell lines and is critical for cell growth and proliferation.”

Allowing for a greater amount of speculation, Mullarky et al. [6] contend that an additional benefit may be derived via diversion of glycolytic flux into serine biosynthesis producing twice as much cytosolic NADH per glucose molecule as compared with the production of pyruvate alone. If true, their hypothesis contends that decreased NADH production in the face of PHGDH knockdown would induce a form of redox stress [6]. The problem is that, with less pyruvate produced as glucose is abstracted from glycolysis at the branch point of 3-phosphoglycerate, cells with elevated serine biosynthesis would need a means to regenerate NAD^+^. How this is accomplished remains unclear. Mullarky et al. [6] contend that perhaps the glycerol phosphate shuttle, which ultimately transfers electrons from cytosolic NADH to FAD^+^ on mitochondrial electron transport chain complex II, may provide a means. In such a situation, the purported pathway could provide an additional benefit to cancer cells by enabling mitochondrial ATP synthesis to occur with less production of reactive oxygen species, passing the electrons from NADH to complex II instead of conventional passage to complex I [6].

Finally, Mullen and DeBerardinis [1] succinctly note that “beyond orchestrating a growth-promoting metabolic phenotype, evidence also suggests that PHGDH, when expressed at high levels, may have properties that prime cells for transformation.” Citing the work of Locasale et al. [8], they point out that overexpression of catalytically active PHGDH but not a hypomorphic mutant in breast epithelial cells induced luminal filling, abnormal nuclear morphology, anchorage-independence, and disturbed cell polarity—all changes associated with cellular transformation in cancerous cells. Such observations, were they to be further assessed, may point to PHGDH overexpression as a means of “enhancing the acquisition” of malignant properties [1].

Across all of these theories, the one thing that becomes collectively clear is that although glycolysis, the TCA cycle, and glutamine metabolism are central to the functioning of normal, and at least to some extent cancerous, cells, they do not act alone [6]. They are part of a much larger metabolic network through which enzymes such as PHGDH act to alter the metabolism of the cell. Bearing this consideration in mind, the oncogenic nature of* PHGDH* amplification likely stems from “a combinatorial effect of pathway flux toward biomass production, changes in redox status, energy metabolism, and possibly some signaling functions” in a manner that “likely varies based on environmental factors, tissue of origin, and cooperating oncogenic mutations” [6]. If the historic progression of PHGDH understanding and subsequent hypotheses to explain its effect has taught us anything, it is that there is much that we still do not know.

## 7. Next Steps: Role as an Oncogene and Argument for Therapeutics

Moving forward, the challenge will be to determine whether or not cancer cells expressing elevated levels of PHGDH require additional serine for growth. Evidence presented throughout this paper speaks to a complicated and uncertain result, for, as we have seen in the work of Locasale et al. [8], higher levels of PHGDH associated with enhanced serine production in melanoma cells—an effect which could be reversed via RNAi depletion of PHGDH. At the same time, work by Possemato et al. [21] found that breast cancer cells with high levels of PHGDH expression are resistant to serine withdrawal. Loss of function with PHGDH knockdown could not be rescued with additional serine [21]. Rather, the authors noted a decrease in the level of aKG produced [21], a finding that was not observed in melanoma cells [8]. A host of mechanistic understandings further underscores the lack of resolution to the question. It is a situation that has led some scholars such as Luo [37] to suggest that purported “addiction” to PHGDH observed in cancer cells might actually represent a set of distinct, albeit variable, “metabolic addictions” with some cells (or cell lines) requiring serine while others require aKG. Evidence from metabolic studies has shown levels of multiple metabolites affected by PHGDH knockdown. Where the discussion becomes interesting is in the correspondingly consistent finding across multiple types of cancer that PHGDH overexpression may directly promote oncogenesis. Using a MCF-10A breast epithelial cell morphogenesis 3D Matrigel assay, Locasale et al. [8] revealed that overexpression of PHGDH increased cellular proliferation rate and disrupted acinar structure such that a change in cellular serine metabolism by way of PHGDH may directly lead to altered phenotypic behavior in a manner that favors transformation [37]. If true, the requirement of additional serine for growth would hold weight, highlighting the need for future studies to determine how this happens and what it means in terms of the broader network of metabolic mechanisms in cancer and the potential for PHGDH as a metabolic oncogene [37].

Ongoing work in the field has begun to consider intersecting pathways such as reported correlations between serine biosynthesis and p73 expression in human lung adenocarcinomas [38] as well as possible regulation of PHGDH by EBV-miR-BART1 in nasopharyngeal carcinomas involving concomitant overexpression of p130Cas and ERBB2 activation [39] or by lack of repression from PKC*ζ* [40]. Recent work by Ma et al. [40] provides compelling evidence that loss of PKC*ζ* function in mice results in increased tumorigenesis and heightened expression of PHGDH as well as PSAT1. Mechanistically, crystal structures of PHGDH dimers reveal phosphorylation sites at Ser55, Thr57, and Thr78 thought to be highly conserved among humans, rats, monkeys, and mice [40]. In prostate cancer, the enzyme has been found to regulate c-Myc phosphorylation [41], and in human intestinal tumor cells, it correlates with caspase-3 [40]. Additional regulation of PHGDH has further been reported in the work of Zhang et al. [42] and Al-Dhaheri et al. [43].

In terms of using PHGDH as a potential therapeutic target, one would need to establish a significant difference between the requirement of the enzyme's activity in cancer and in normally proliferating cells [15]. Over the last 30 years, studies have satisfied this objective, strongly implicating PHGDH as an attractive drug target in the subset of tumors that amplify and overexpress its gene [37]. The important determination will lie in whether or not a sufficient therapeutic window exists, given that serine biosynthesis operates in all cells. Mitigating this concern, Luo [37] maintains that PHGDH inhibition remains a viable cancer therapeutic target for two reasons: (1) a PHGDH inhibitor designed not to cross the blood-brain barrier would not interfere with serine homeostasis in the central nervous system, avoiding potential neurological effects reported for known PHGDH mutations in humans, and (2) serine deficiency disorders can be treated by exogenous serine supplement, whereas tumors' addiction to PHGDH might not be associated with serine flux. Thus, a PHGDH inhibitor nonpermeable to the central nervous system coupled with serine supplement (if it can be designed) may provide the therapeutic index needed to selectively target tumorigenic cells [37]. Considerable work remains to be done before such a result could be achieved, particularly to elucidate the complex pathway and underlying control seemingly involved with PHGDH. As exemplified in recent work by Chen et al. [44], the results of such studies are far from clear and may ultimately depend on context- and stage-specific factors. In light of these considerations and the complexities revealed by previous attempts to understand metabolic PHGDH function using cancer cell lines and tumor xenografts, it is thought that knock-in models in mice may provide an alternative choice as an animal model with a less confounded genetic background [44]. Regardless of the specific model and experimental methodology used, work remains to be done as researchers continue the ongoing pursuit of a potential therapeutic target and putative metabolic oncogene.

## Figures and Tables

**Table 1:** Summary of major contributions to the understanding of phosphoglycerate dehydrogenase (PHGDH) in cancer.

Study	Year	Type of cancer	Contribution
Snell et al. [16–20]	1984–1988	Hepatoma (rat)	(i) Elevation of PHGDH
			(ii) Did not respond to changes in dietary protein level
			(iii) Correlated with increased rate of serine biosynthesis
			(iv) Correlated with tumorigenic rate of growth
			(v) Associated with neoplastic transformation and progression
			(vi) Corresponding increases in serine hydroxymethyltransferase activity, absence of serine dehydratase and serine aminotransferase (Figure 2)
		Sarcoma (rat)	(i) Consistent effects
		Colon carcinoma (human)	(i) Consistent effects

Cho et al. [22]	2000	Leukemia (human)	(i) Detectable 2.1 kb and 710 bp mRNA PHGDH transcripts
		T-cell lymphoblastic lymphoma (human)	(i) Detectable 2.1 kb and 710 bp mRNA PHGDH transcripts
		Colon adenocarcinoma (human)	(i) Detectable 2.1 kb and 710 bp mRNA PHGDH transcripts
		Epithelioid carcinoma (human)	(i) Detectable 2.1 kb and 710 bp mRNA PHGDH transcripts
		Lymphoma (murine)	(i) Detectable 2.1 kb and 710 bp mRNA PHGDH transcripts

Pollari et al. [23]	2011	Bone metastatic breast cancer	(i) Enhanced serine production
			(ii) Stimulation of osteoclastogenesis
			(iii) Genetic upregulation of PHGDH, PSAT1, and PSPH (Figure 2)
			(iv) Association with shorter time to relapse, reduced survival time, and several “clinically relevant features”

Possemato et al. [21]	2011	Breast cancer	(i) *PHGDH* gene exists in a region of chromosome 1p (1p12) amplified in several types of cancer
			(ii) Elevated protein expression in estrogen receptor-negative breast cancer, relative to estrogen receptor-positive tumors approx. 68% mRNA and 70% protein elevations
			(iii) Protein levels were elevated in estrogen receptor-negative cells lacking genetic copy number gains
			(iv) Drives glucose-originating flux through the biosynthetic serine pathway
			(v) Serine production is not the only important role for PHGDH in cancer cells → parallel increase in PSAT1 expression and conversion of glutamate to aKG

Locasale et al. [8]	2011	Melanoma	(i) “Substantial fraction” of glycolytic flux diverted to serine production
			(ii) *PHGDH* gene exists in a region of chromosome 1p (1p12) amplified in several types of cancer
			(iii) Found localized amplification of *PHGDH* within the coding region of the gene
			(iv) Elevated protein expression in human melanoma cells
		Breast cancer	(i) High protein expression associated with triple-negative and basal subtypes but did not associate with metastasis or tumor size (contrary to previous results)

Liu et al. [24]	2013	Astrocytoma/glioma	(i) Elevation in brain tissue not normally expressing PHGDH
			(ii) Correlated with progressively advanced tumor grade
			(iii) Stabilizing binding interaction with oncogenic transcription factor FOXM1, induction of a series of known oncogenes

Jing et al. [25]	2013	Cervical cancer	(i) Elevated protein expression in squamous cell carcinoma
			(ii) Associated with tumor progression, stage, and size

Noh et al. [26]	2014	Triple-negative breast cancer	(i) Basal marker-positive patients exhibited increased protein expression relative to basal marker-negative patients
			(ii) Protein expression was high in patients with mixed basal-like subtypes; 89.3% of mixed subtypes showed partial expression of basal markers
